# Life insurance salespeople linking work stressors to proactive behaviors by passion: Servant leadership as a moderator

**DOI:** 10.3389/fpsyg.2022.977981

**Published:** 2022-10-28

**Authors:** Aijun Weng, Lingjun Zhou, Fufu Sun

**Affiliations:** College of Business, Shanghai University of Finance and Economics, Shanghai, China

**Keywords:** work stressors, proactive behaviors, work passion, servant leadership, self-determination theory

## Abstract

As the main sales force of life insurance companies, salespeople have accounted for more than 50% of life insurance sales channels over the years, playing a pivotal role in the development of the industry. Since the adoption of the model of employment at an agency, the commission income of life insurance salespeople has largely relied on their sales volume, which requires employee proactivity under a great number of stressors. However, because previous studies have analyzed stressors in a single dimension, our understanding of the relationship between work stressors and proactive behaviors remains limited. Applying self-determination theory, we investigated differential relationships between challenge/hindrance stressors and proactive behaviors, which were expected to be mediated by passion and moderated by servant leadership. In the sample of 332 insurance salespeople, there was a positive (negative) relationship between challenge (hindrance) stressors and proactive behaviors. In addition, passion mediated the relationship between stressors and proactive behaviors, and servant leadership moderated the relationship between stressors and passion. Theoretical and practical implications are discussed.

## Introduction

Nowadays, it is increasingly difficult to run a modern enterprise in a traditional way. The relationship between an enterprise and its employees is more like a partnership rather than an exchange relationship in which the enterprise pays employees for their mental or physical labor, as now employee proactive behavior weighs heavily into organizational success (Frese et al., 2007; Parker and Collins, 2010). Especially, sales-driven organizations like insurance companies view salespeople’s proactive behaviors as essential for gaining a competitive advantage because these individuals are the main channel for sales performance (The Economics, 2020). Proactive behavior, which is considered self-initiated, anticipatory action aimed at changing either the situation or oneself (Grant and Ashford, 2008; Bindl and Parker, 2011), has been shown to benefit both organizations and individuals by leading to better socialization (Ellis et al., 2015), greater sales performance (Crant, 1995), and more innovative behavior (Seibert et al., 2001). As a result, employee proactivity has become a burgeoning topic among both scholars and practitioners.

Although extensive attention has been paid to the relationship between work stressors and proactive behaviors, the findings are still far from certain. On one hand, scholars have argued that stressors can impede employee proactive behaviors by depleting the available resources (e.g., Bande et al., 2019; Xu et al., 2019) or by inducing a negative mood (e.g., Lee et al., 2018). On the other hand, work stressors have been proven to increase proactive behaviors, for example, by fueling higher work motivation (e.g., Lepine et al., 2005; Ohly et al., 2006) and openness to change (e.g., Bao et al., 2019).

Drawing on self-determination theory (SDT) (Deci and Ryan, 2012) and the challenge-hindrance stress model (CHM), we aim to address this controversial issue by distinguishing work stressors as either challenge stressors and hindrance stressors (Cavanaugh et al., 2000) and investigating how each type affects employee proactive behaviors through the mediating effect of passion. SDT posits that the satisfaction of three fundamental human needs (i.e., autonomy, competence, and relatedness) determines the quality of individuals’ motivation (Deci and Ryan, 1985; Gagné and Deci, 2005). Passion, anchored in the STD perspective, is a pivotal, motivational mechanism in linking contextual characteristics and human behaviors (Vallerand et al., 2003; Vallerand and Young, 2014). Harmonious passion refers to an internalized motivational tendency to proactively engage in activities, whereas obsessive passion is non-self-determined, constraining individuals to attend activities (Seguin-Levesque et al., 2003; Vallerand et al., 2003). Hence, it is plausible to argue that when employees appraise the environment as full of challenge stressors, which signal opportunities to achieve desirable outcomes (needs satisfaction), harmonious passion emerges and they are motivated to engage in proactive behaviors. In contrast, when employees view stressors as hindrances, which are controlled and may thwart personal growth and goal attainment (needs dissatisfaction), the obsessive passion is developed, impeding proactivity (Vallerand et al., 2003; Lepine et al., 2005; Podsakoff et al., 2007).

We further propose that the effects of challenge and hindrance stressors on employee passion vary with the extent of employees’ perception of servant leadership in the organization. As key decision-makers and resource allocators, team leaders receive leeway to influence employees’ psychological processing (Zhang et al., 2014; Ciobanu et al., 2019; He et al., 2020). Servant leadership, with the central premise of satisfying subordinates’ basic needs, “comes to recognize the three basic psychological needs of SDT and to contribute to their satisfaction” (van Dierendonck, 2011; Chiniara and Bentein, 2016; Brière et al., 2021). In a nod to these ideas, we consider servant leadership as boundary conditions and expect that this key contextual factor may amplify or mitigate the relationship between work stressors and employee passion.

We test our integrative model by focusing on life insurance salespeople. First, life insurance salespeople can offer us a pure look at proactive behaviors. Since they work without a basic salary, they depend on their proactivity to reach better sales performance, thus earning higher pay. Second, due to increasing stressors in the workplace and the stressful nature of sales positions (Boles et al., 1997), pay-for-performance incentives may no longer serve as the single magic bullet for achieving desirable organizational outcomes: at the end of 2021, 80% growth was brought by 20% proactive salespeople, and the total number of life insurance salespeople has dramatically fallen by 34% from its peak of 9.12 million in late 2019 (China Banking and Insurance Regulatory Commission, 2022). Taken together, these facts indicate the importance of deepening our understanding of life insurance salespeople’s proactive behavior as they navigate through work stressors for both organizational and individual success.

By examining how work stressors are linked to employee proactive behaviors through the mediating effect of passion, this manuscript provides several indepth contributions to the literature. Firstly, despite growing interest, no study yet has integrated the mixed findings on the influences that work stressors exert on employee proactive behaviors. Drawing on SDT, we provide an integrated model clarifying how challenge/hindrance stressors affect proactive behaviors in positive/negative ways through distinct mechanisms. Secondly, by explicitly analyzing harmonious/obsessive passion as the underlying mechanism, we respond to calls from Fritz and Sonnentag (2009) to investigate more person-related motivational factors that fuel or hinder proactive behaviors. Third, our study advances SDT by highlighting the interaction between stressors and servant leadership. Since hindrance stressors inevitably appear in the work environments of insurance salespeople (Boles et al., 1997; Bande et al., 2019), our study provides a new insight into the moderating effect of servant leadership, which may support salespeople to cultivate more “beneficial” passion and behave more proactively. Finally, based on the validated model and mechanism, we offer a rational solution to improve the work situation of life insurance salespeople by guiding members to appraise work stressors in a more positive way and by modestly enhancing servant leadership. Hence, proactive behaviors will be facilitated to the benefit of both organizations and individual employees.

## Theoretical basis and research hypotheses

### Work stressors and proactive behaviors

The literature generally defines employees’ proactive behaviors as spontaneous individual actions with the purpose of changing themselves or the current environment (Crant, 2000). Employees can behave proactively across several domains, such as voice (Morrison, 2011), feedback seeking (Ashford et al., 2003), job crafting (Tims et al., 2012), and socialization (Ashford and Black, 1996). All these actions have in common that they are self-initiated, involving “the individual actively taking control and ‘making things happen”’ (Parker et al., 2010).

Since proactive behaviors are generally linked to desirable outcomes, their antecedents have received extensive attention. For instance, several studies have explored the predictive role of work stressors, defined as objective external conditions or events that create stressful demands on or threats to individuals (Lazarus, 1990). As noted above, empirical research has revealed inconsistent results, with some studies showing a positive relationship (e.g., Lepine et al., 2005; Ohly et al., 2006; Babalola et al., 2021) and others showing a negative relationship (e.g., Lee et al., 2018; Bande et al., 2019; Xu et al., 2019) between stressors and proactive behaviors. As Cavanaugh et al. (2000) indicated, these mixed findings may due to the fact that work stressors, which have both positive and negative dimensions, were considered as a single category, canceling out their differential effects.

Applying this line of research, we adopt a distinction between challenge stressors and hindrance stressors and employ SDT to deeply investigate the separate roles of the two in affecting proactive behaviors. According to SDT, an individual’s motivation is determined by the satisfaction of fundamental human needs (Gagné and Deci, 2005). When individuals perceive that they are able to satisfy their needs for autonomy, competence, and relatedness within social environments, they see opportunities for favorable consequences and are more likely to proactively take action. In contrast, when individuals view environments as thwarting, which may undermine their basic needs, their motivation might be muzzled (Ryan and Deci, 2000; Gagné and Deci, 2005).

We argue that challenge stressors facilitate employee proactive behaviors, whereas hindrance stressors do the opposite. Work stressors are objective environmental factors (e.g., job demands), but they can be subjectively experienced as challenges or hindrances (Mitchell et al., 2019). Moreover, although challenge and hindrance stressors should be viewed as two independent constructs, they are not always mutually exclusive: a stressor can be simultaneously considered as both a challenge and hindrance (Horan et al., 2020). Cavanaugh et al. (2000) posited that challenge stressors encompass factors perceived as manageable. Individuals consider challenge stressors as opportunities for personal growth and development, which cater to their fundamental needs. Therefore, although challenge stressors may inevitably bring psychological or physical discomfort, individual may still have strong motivations to actively cope with them because of the perception of favorable outcomes (Cavanaugh et al., 2000; Liu et al., 2022). In contrast, hindrance stressors encompass factors perceived as beyond one’s control. Therefore, employees may feel threatened since their need for personal growth is thwarted, and they are more likely to turn to withdrawal and avoidance (Wallace et al., 2009) instead of engaging in proactive behaviors.

Evaluating the relationship between stressors and proactive behaviors is particularly important for life insurance salespeople. At first, proactive behaviors were found to play an essential role in sales performance. Porath and Bateman (2006) suggested that proactive behaviors can effectively predict sales quota achievement, and several empirical studies showed they can increase sales performance (e.g., Murphy and Coughlan, 2018; Varela et al., 2019). In addition, since the sales environment is naturally full of stressors (Boles et al., 1997; Bande et al., 2019), including the no-basic-salary compensation structure, insurance salespeople may weigh whether or not to proactively take action in a more subtle way. That is, they may evaluate work stressors as challenges because successfully coping with those stressors signals opportunities for needs satisfaction such as higher performance and career advancement. Thus, salespeople may voluntarily engage in proactive behaviors. To the contrary, they may also evaluate work stressors as hindrances because they perceive that they are unable to satisfy their needs within unmanageable environments, leading to less proactivity. Within the given frame of reference, we hereby propose Hypotheses 1 and 2:

Hypothesis 1: Challenge stressors are positively related to proactive behaviors.

Hypothesis 2: Hindrance stressors are negatively related to proactive behaviors.

### The mediating role of passion in the work stressors-proactive behavior link

Furthermore, we argue that passion can function as a mechanism underlying the association between work stressors and proactive behaviors. Passion, as Vallerand (2010) described, is a motivational construct that refers to an individual inclination toward self-defining activities. Unlike intrinsic and extrinsic motivation, passion is relatively stable and encompasses both cognitive and affective components (Ho and Astakhova, 2018), with stronger links to jobs or specific activities (Vallerand et al., 2003; Liu et al., 2011).

Passion can be further differentiated into two categories according to how the job is internalized into one’s identity (Vallerand et al., 2003; Vallerand and Young, 2014). Harmonious passion stems from autonomous internalization and is accompanied by positive emotions. Obsessive passion, however, stems from pressures and instrumental outcomes (e.g., rewards, punishments, or promotion) and is accompanied by negative emotions (Vallerand et al., 2003). Recent evidence has pointed to passion as a mediating variable within a range of relationships linking environmental factors and individual outcomes. For example, Liu et al. (2011) posited that harmonious passion acts as a mediator between team support for autonomy and members’ creativity. Moreover, Xiao et al. (2020) explored both harmonious and obsessive passion as mediating variables between temporal leadership and employees’ innovative behaviors.

We expected harmonious passion (obsessive passion) to play an important role in translating challenge stressors (hindrance stressors) into employee proactive behaviors for several reasons. First, individuals’ perception of work stressors can generate different types of passion. Building on SDT, when individuals positively appraise work environment characteristics (e.g., appraise stressors as challenges), they perceive their activity as self-determined and develop a harmonious passion. Contrarily, when individuals appraise stressors as hindrances, they perceive their activity as forced and their needs are difficult to satisfy, leading to the emergence of obsessive passion.

Second, since the fundamental component of proactive behavior is that employees intentionally take actions to cope with and change their work environment rather than passively accept it (Parker and Collins, 2010), activating proactive behaviors requires strong motivation. Hence, from the perspective of cognition, it is rational to expect that individuals with harmonious passion are inclined to take more proactive actions because they autonomously internalize a superior motivation (Vallerand et al., 2003; Liu et al., 2011), whereas individuals with obsessive passion exhibit less proactivity because they believe that they are controlled and forced.

Third, passion also brings about positive or negative affect (Vallerand et al., 2003; Mageau et al., 2009). With harmonious passion, individuals approach work activities with enthusiasm and joy, allowing them to proactively and fully engage in those activities (Vallerand and Young, 2014; Ho and Astakhova, 2018). In contrast, obsessive passion leads individuals to experience negative affect such as anxiety and nervousness, inhibiting their engagement in proactive behaviors. Hypotheses 3 and 4 are formulated as follows:

Hypothesis 3: Harmonious passion mediates the positive relationship between challenge stressors and proactive behaviors.

Hypothesis 4: Obsessive passion mediates the negative relationship between hindrance stressors and proactive behaviors.

### Servant leadership as the moderator

SDT emphasizes that the satisfaction of fundamental needs derives from the dialectic between individuals and their environmental context (Ryan and Deci, 2000; Gagné and Deci, 2005). Leadership plays an integral and central part of organizational environments and has an essential influence on employees’ needs satisfaction (Liao et al., 2015; Brière et al., 2021). Therefore, previous empirical studies claimed that leadership is an important factor affecting employees’ work passion. For example, Ho and Astakhova (2020) argued that charismatic leadership is positively associated with employees’ harmonious passion and contingent reward leadership is negatively associated with employees’ obsessive passion.

Servant leadership is characterized by a focus on employee growth and empowerment, that prioritizes individuals’ personal growth and career development (Greenleaf, 1977; Friedman and Mizrachi, 2022). Unlike other forms of leadership, servant leadership has a fundamental bottom-top characteristic: explicit attention on meeting subordinates’ needs (Brière et al., 2021). Specifically, by providing developmental support (Chen et al., 2015), enhancing followers’ well-being (van Dierendonck, 2011), and offering emotional resources (Barbuto and Wheeler, 2006), servant leaders may have an impact on the emergence of passion from work stressors.

To further ensure that our model could be valid, we conducted pilot interviews among life insurance salesperson. The main source of data was audio-recorded interviews collected in Aug 2020. We conducted 20 interviews with semi-structured, open-ended protocol that focused on salesperson’s daily works, their motivation in proactivity, their feeling among work stressors, and the role of leader. Among 20 informants, 65% are male and 35% are female; half subordinates and half middle managers. Each of interview lasted 30–45 min. As a result, almost all informants reported that their leaders play a key role in amplifying the positive impact and attenuating the negative impact from stressors. As one informant mentioned, “stressors are tough…but my leader care about my needs not only among workplace but also among my life…I should show my persistence and proactivity to my leader in return.”

Thus, this study argues that servant leadership may be a supportive or compensative component of context, conducive to the stressors-passion relationship. Since the development of one’s career largely depends on sales performance, and sales performance largely depends on proactive behaviors (Murphy and Coughlan, 2018; Bande et al., 2019; Varela et al., 2019), life insurance salespeople may deem stressors as challengeable opportunities, internalizing motivation, and generating harmonious passion. In this situation, servant leaders, who consider employee needs (van Dierendonck, 2011), may provide the conditions for their subordinates to develop harmonious passion.

At the same time, however, it is also possible to image that the specific environments of life insurance companies may convey pressure and uncertainty to salespeople. Life insurance salespeople may perceive that they are under external control, for example, if they are forced to achieve a sales target, and this can generate obsessive passion. In this situation, servant leadership can play a compensative role, satisfying subordinates’ needs by providing emotional resources or encouraging subordinates to foster useful skills (Eva et al., 2018). Thus, with support from servant leaders, life insurance salespeople may develop less obsessive passion even they still view work stressors as hindrances. Hypotheses 5 and 6 are posited:

Hypothesis 5: Servant leadership moderates the positive relationship between challenge stressors and harmonious passion, such that the relationship is more positive when servant leadership is higher.

Hypothesis 6: Servant leadership moderates the positive relationship between hindrance stressors and obsessive passion, such that the relationship is less positive when servant leadership is higher.

Based on Hypotheses 5 and 6, this manuscript further proposes that servant leadership moderates the mediating effect of harmonious/obsessive passion in the first stage. That is, (1) when insurance salespeople simultaneously face challenge stressors and perceive strong servant leadership, they are more likely to experience harmonious passion, which will amplify the facilitation of their proactive behaviors; and (2) when insurance salespeople simultaneously face hindrance stressors and perceive strong servant leadership, they are less likely to experience obsessive passion, which will attenuate the inhibition of proactive behaviors. Then, Hypotheses 7 and 8 are as shown below:

Hypothesis 7: Servant leadership moderates the mediating effect of harmonious passion in the first stage. That is, the indirect effect will be stronger (weaker) when servant leadership is high (low).

Hypothesis 8: Servant leadership moderates the mediating effect of obsessive passion in the first stage. That is, the indirect effect will be weaker (stronger) when servant leadership is high (low).

The overall theoretical model is summarized in the following figure.

## Materials and methods

### Participants and procedure

The data were collected through a questionnaire survey. The research sample consisted of the life insurance salespeople at life insurance company in the Shanghai market. The survey was carried out with the consent of the person in charge of the company. To ensure, to the extent possible, that the research results were not affected by the same source bias, the questionnaire was administered in two parts with an interval of 2 weeks in between. All questionnaire responses were collected on the spot.

The first part of the questionnaire asked about demographic information, challenge stressors, hindrance stressors, servant leadership, harmonious passion, and obsessive passion. A total of 400 questionnaires were distributed, and 375 responses were collected. The valid collection rate was 93.75%. The second questionnaire measured employees’ proactive behaviors. A total of 375 questionnaires were distributed, and 350 responses were collected, with a valid collection rate of 93.33%. Finally, we integrated the data of 350 valid questionnaire responses from both times and excluded missing and invalid responses, such as those with many missing values or repeated values, obvious regularity, or no match across the two stages. In the end, 332 valid responses were obtained, with a final valid rate of 83%.

Among the 332 employees, 61.4% were male and 38.6% were female; 11.5% were under 25 years old, 46.1% (the largest age group) were aged 25–30, 31% were 31–40, 6.9% were 41–50, and 4.5% were 51 or above; 53% (the largest education group) had a college degree, 29.8% had graduated from high school or a secondary specialized school, 16.9% held a bachelor’s degree, and 3% held a master’s degree or above.

### Measurement scale

Stressors: The scale developed by LePine et al. (2016) was used to examine the influence of charismatic leadership on the relationship between follower stress and job performance for the measurement of challenge stressors and hindrance stressors. The scale has been validated by previous studies as reliable, with 10 challenge stressors and 10 hindrance stressors. This scale and all scales below adopt 7-point scoring from 1 “completely disagree” to 7 “completely agree.” In this study, the consistency reliability coefficient of challenge stressors was 0.86, and that of hindrance stressors was 0.93.

Servant leadership: The scale developed by Liden et al. (2014) was used to measure servant leadership. It has been verified to have good reliability in research on the impact of servant leadership and service culture on individual and organizational performance. There are 7 items in total. In this study, the consistency reliability coefficient was 0.89.

Work passion: To measure harmonious passion and obsessive passion, the scale developed by Sirén et al. (2016) was employed. The reliability of the scale has been validated in previous studies that verified the moderating effect of harmonious passion and obsessive passion on the relationship between CEOs’ change-oriented leadership and organizational performance. The scale contains 7 items on harmonious passion and 7 items on obsessive passion. In this study, the consistency reliability coefficient of harmonious passion was 0.93, and that of obsessive passion was 0.95.

Proactive behaviors: The measurement of proactive behaviors adopted the scale developed by Griffin et al. (2007) when studying proactive behaviors in uncertain and interdependent contexts. It is a classic scale for proactive behaviors used by many studies. It contains 6 items. In this study, the consistency reliability coefficient was 0.72.

Control variables: Employees’ age, gender, length of time working under the current leader, and educational background were taken as control variables because previous studies have found that such demographic characteristics have an impact on proactive behaviors.

### Analysis

SPSS 23.0 and AMOS 21.0 were used to analyze the data. The discriminant validity of the selected variables was analyzed by confirmatory factor analysis (CFA). Harman’s single-factor test was used to investigate the common method variance. Then, descriptive statistical analysis and correlation analysis were performed on the variables. A mediation test and moderation hypothesis test were carried out, as well as a moderated mediation test.

## Results

### Confirmatory factor analysis

AMOS21.0 was used for the CFA. The nested model method was applied to test the discriminant validity by comparing the fit of each model through CFA. For the five variables of challenge stressors, hindrance stressors, servant leadership, harmonious passion, and obsessive passion, the goodness of fit of the four-, three-, two-, and single-factor models were significantly worse than that of the five-factor model, which is sufficient to show that the challenge stressors, hindrance stressors, servant leadership, harmonious passion, and obsessive passion that this study focuses on have significant discriminant validity. The results are summarized in Table 1.

**TABLE 1:** Results of discriminant validity test.

Model	Factor structure	χ^2^	df	CFI	IFI	SRMR
Base model	Five factors	1146.98	99	0.84	0.78	0.13
Model 1	Four factors	1389.25	103	0.80	0.74	0.15
Model 2	Three factors	2296.89	106	0.66	0.57	0.20
Model 3	Two factors	2698.00	109	0.60	0.50	0.20
Model 4	One factor	4424.24	110	0.34	0.18	0.29

In the four-factor model, challenge stressors and hindrance stressors are combined. In the three-factor model, challenge stressors and hindrance stressors are combined; servant leadership and obsessive passion are combined. In the two-factor model, challenge stressors and hindrance stressors are combined; harmonious passion, obsessive passion and servant leadership are combined.

### Test of common method variance

Unrotated principal component analysis was performed. The results of exploratory factor analysis showed that the first factor accounted for 28.27% of the total interpretation rate of the factors, which is not an absolute proportion of the total interpretation rate. Therefore, the common method variance did not have a great impact on the theoretical model of this study.

### Descriptive statistical results

As shown in Table 2, and as theoretically supported and expected, in Hypotheses 1 and 2, challenge stressors were positively correlated with proactive behaviors (*r* = 0.26, *P* < 0.001), and hindrance stressors were negatively correlated with proactive behaviors (*r* = –0.30, *P* < 0.001). Moreover, challenge stressors were positively correlated with harmonious passion (*r* = 0.17, *P* < 0.01); harmonious passion was positively correlated with proactive behaviors (*r* = 0.15, *P* < 0.01); hindrance stressors were positively correlated with obsessive passion (*r* = 0.27, *P* < 0.001); and obsessive passion was negatively correlated with proactive behaviors (*r* = –0.22, *P* < 0.001). Thus, the main hypotheses of this study are preliminarily supported.

**TABLE 2:** Descriptive statistics and correlation analysis results.

Variable	*M*	SD	1	2	3	4	5	6	7	8	9	10
1. Age	31.45	7.84	–									
2. Gender^a^	1.61	0.49	–0.05	–								
3. Time of working under the current leader	0.47	1.05	0.22***	0.07	–							
4. Education^b^	2.88	0.68	–0.10	–0.04	–0.19***	–						
5. Challenge stressors	5.29	0.78	–0.16*	0.06	–0.03	–0.05	–					
6. Hindrance stressors	3.80	1.24	–0.04	0.04	–0.03	0.06	0.30***	–				
7. Servant leadership	5.15	1.15	0.11	0.12*	0.28***	–0.16**	0.11*	–0.22***	–			
8. Harmonious passion	4.36	0.92	0.01	0.09	0.07	–0.09	0.17**	–0.18***	0.78***	–	–	
9. Obsessive passion	3.74	1.46	0.00	0.00	0.16**	–0.07	0.21***	0.27***	0.30***	0.47***	0.38***	–
10. Proactive behaviors	3.95	0.51	–0.18***	–0.01	–0.27***	–0.09	0.26***	–0.30***	0.09	**0.16** **	0.15**	–0.22***

Number of employees: 332.

^a^Gender: 1 = female, 2 = male.

^b^Education: 1 = junior high school and below, 2 = secondary specialized school or high school, 3 = college, 4 = bachelor’s degree, 5 = master’s degree, 6 = doctoral degree. *p < 0.05, **p < 0.01, ***p < 0.001, two-tailed test.

### Hypotheses testing

#### Main effect testing

SPSS 23.0 was used to perform hierarchical regression. Specifically, (1) with harmonious passion as the dependent variable and employees’ age, gender, length of time working under the current leader, and educational background as the control variables (Model 1 in Table 3), challenge stressors were put into the regression equation to test the influence on harmonious passion (Model 2 in Table 3). Model 2 in Table 3 shows that challenge stressors had a significant positive impact on harmonious passion (*B* = 0.20, *P* < 0.01). (2) With proactive behaviors as the dependent variable and employees’ age, gender, length of time working under the current leader, and educational background as the control variables (Model 1 in Table 4), harmonious passion was put into the regression equation to test the influence on proactive behaviors (Model 3 in Table 4). The results revealed that harmonious passion had a significant positive influence on proactive behaviors (*B* = 0.09, *P* < 0.01). (3) With obsessive passion as the dependent variable and employees’ age, gender, length of time working under the current leader, and educational background as the control variables (Model 1 in Table 5), hindrance stressors were put into the regression equation to test the influence on obsessive passion (Model 2 in Table 5), and the results showed that they had a significant positive effect on obsessive passion (*B* = 0.33, *P* < 0.001). (4) With proactive behaviors as the dependent variable and employees’ age, gender, length of time working under the current leader, and educational background as the control variables (Model 1 in Table 4), obsessive passion was put into the regression equation to test the influence on proactive behaviors (Model 6 in Table 4). Obsessive passion had a significant negative impact on proactive behaviors (*B* = –0.07, *P* < 0.001).

**TABLE 3:** Hierarchical regression analysis results (Harmonious passion).

	Harmonious passion											
Variable	Model 1		Model 2		Model 3		Model 4		Model 5		Model 6	
	*B*	*t*	*B*	*t*	*B*	*t*	*B*	*t*	*B*	*t*	*B*	*t*
Intercept	4.40	12.02	3.22	6.15	0.66	1.94	1.01	3.07	1.09	2.48	1.30	2.98
Age	–0.00	–0.11	0.00	0.37	0.00	–0.95	0.00	–0.57	0.00	–0.46	0.00	–0.54
Gender	0.15	1.48	0.14	1.35	–0.01	–0.22	–0.04	–0.68	0.07	0.93	0.07	0.84
Time of working under the current leader	0.05	0.97	0.05	1.03	–0.13	–3.98	–0.12	–3.93	0.03	0.65	0.00	0.07
Education	–0.10	–1.36	–0.09	–1.18	0.02	0.47	0.01	0.27	–0.09	–1.55	–0.10	–1.65
Challenge stressors			0.20**	3.11	0.09	2.21	0.04	1.15	0.03	0.51	0.05	1.04
Servant leadership					0.65***	22.92	0.63***	23.23				
Challenge stressors × servant leadership							0.16***	6.19				
*F*	1.50		9.65**		525.09***		38.36***		205.67***		9.88**	
*R* ^2^	0.02		0.05		0.64		0.67		0.42		0.43	
Δ*R*^2^	0.02		0.03		0.59		0.04		0.04		0.02	

Number of employees: 332, **p < 0.01, ***p < 0.001.

**TABLE 4:** Hierarchical regression analysis results (Proactive behaviors).

	Proactive behaviors													
Variable	Model 1		Model 2		Model 3		Model 4		Model 5		Model 6		Model 7	
	
	*B*	*t*	*B*	*t*	*B*	*t*	*B*	*t*	*B*	*t*	*B*	*t*	*B*	*t*
Intercept	4.64	23.82	3.77	13.72	4.25	18.41	3.55	12.31	5.08	25.70	4.93	23.93	5.21	25.47
Age	–0.01**	–2.61	–0.01	–1.98	–0.01**	–2.63	–0.01	–2.05	–0.01**	–2.93	–0.01**	–2.82	–0.01**	–3.02
Gender	0.00	–0.04	–0.01	–0.26	–0.02	–0.29	–0.02	–0.44	0.01	0.24	–0.01	–0.10	0.01	0.18
Time of working under the current leader	–0.13***	–4.89	–0.13***	–4.97	–0.13***	–5.11	–0.13***	–5.13	–0.13***	–5.28	–0.11***	–4.34	–0.12***	–4.86
Education	–0.12**	–2.90	–0.11**	–2.70	–0.11**	–2.70	–0.10**	–2.56	–0.10**	–2.73	–0.12**	–3.13	–0.11**	–2.88
Challenge stressors			0.15***	4.37			0.13***	3.93						
Hindrance stressors									–0.13***	–6.21			–0.11***	–5.35
Harmonious passion					0.09**	3.04	0.07*	2.39						
Obsessive passion											–0.07***	–3.77	–0.04**	–2.24
*F*	10.17***		19.11***		9.21**		5.71*		38.62***		14.24***		4.99*	
*R* ^2^	0.11		0.16		0.14		0.17		0.21		0.15		0.22	
Δ*R*^2^	0.11		0.05		0.02		0.02		0.09		0.04		0.12	

Number of employees: 332, *p < 0.05, **p < 0.01, ***p < 0.001.

**TABLE 5:** Hierarchical regression analysis results (Obsessive passion).

	Obsessive passion											
Variable	Model 1		Model 2		Model 3		Model 4		Model 5		Model 6	
	*B*	*t*	*B*	*t*	*B*	*t*	*B*	*t*	*B*	*t*	*B*	*t*
Intercept	4.24	7.36	3.08	5.19	0.52	0.78	1.13	1.62	–0.65	–1.03	0.07	0.11
Age	–0.01	–0.75	–0.01	–0.63	–0.01	–0.97	–0.01	–1.14	–0.01	–1.01	–0.01	–1.01
Gender	–0.05	–0.29	–0.09	–0.54	–0.21	–1.40	–0.21	–1.41	–0.18	–1.29	–0.18	–1.31
Time of working under the current leader	0.23**	2.87	0.23**	3.09	0.11	1.55	0.13	1.78	0.21**	3.12	0.21	3.17
Education	–0.10	–0.83	–0.13	–1.15	–0.06	–0.54	–0.08	–0.74	–0.12	–1.21	–0.13	–1.33
Hindrance stressors			0.33***	5.39	0.42***	7.09	0.36***	5.66	0.34***	6.25	0.27***	4.81
Servant leadership					0.46***	6.82	0.41***	6.09				
Hindrance stressors × servant leadership							0.12**	2.83				
*F*	2.51*		29.07***		39.83***		8.03**		104.03***		12.84***	
*R* ^2^	0.03		0.11		0.22		0.24		0.33		0.35	
Δ*R*^2^	0.03		0.08		0.11		0.02		0.22		0.03	

Number of employees: 332, *p < 0.05, **p < 0.01, ***p < 0.001.

#### Test of the mediating effect of work passion

First, a three-step hierarchical regression method was used to verify the mediating effect of positive team emotion (Baron and Kenny, 1986). That is, the relationship between the independent variable and dependent variable were analyzed, followed by the relationship between the mediating variable and dependent variable. If both relationships were significant, the relationships among the independent variable, mediating variable, and dependent variable were also analyzed. After the mediating variable was added, if the relationship between the mediating variable and dependent variable was significant but the relationship between the independent variable and dependent variable was no longer significant, it was considered a fully mediating variable; if the effect size of the relationship between the independent variable and dependent variable became smaller but was still significant, it meant that there was partial mediation.

Model 2 in Table 4 shows that challenge stressors were positively correlated with proactive behaviors (*B* = 0.15, *P* < 0.001). Next, Model 3 shows that harmonious passion was positively correlated with proactive behaviors (*B* = 0.09, *P* < 0.01). As shown in Model 4, after harmonious passion was added, its regression coefficient, *B* = 0.07, *P* < 0.05, and the effect size between challenge stressors and proactive behaviors became smaller but were still significant (*B* = 0.13, *P* < 0.001), indicating that harmonious passion played a mediating role between challenge stressors and proactive behaviors. Based on the above, Hypothesis 3 is verified. In addition, this study used Bootstrapping for interval estimation, and the confidence interval was [0.001, 0.03], excluding 0. Hence, the mediation effect is further verified.

Model 5 in Table 4 shows that hindrance stressors were negatively correlated with proactive behaviors (*B* = –0.13, *P* < 0.001). Model 6 shows that obsessive passion was negatively correlated with proactive behaviors (*B* = –0.07, *P* < 0.001). As shown in Model 7, after obsessive passion was added, its regression coefficient, *B* = –0.04, *P* < 0.05, and the effect size between hindrance stressors and proactive behaviors became smaller but were still significant (*B* = –0.11, *P* < 0.001), which indicates that obsessive passion played a mediating role between hindrance stressors and proactive behaviors. Based on the above, Hypothesis 4 is verified. In addition, this study used Bootstrapping for interval estimation, and the confidence interval was [–0.03, –0.003], excluding 0. The mediation is further verified.

#### Test of the moderating effect of servant leadership

When testing the moderating effect of servant leadership on the relationship between challenge stressors and harmonious passion, as well as the relationship between hindrance stressors and obsessive passion, in order to avoid the problem of collinearity, we first performed centralized processing on challenge stressors, hindrance stressors, and servant leadership. According to the results of centralized processing, the interaction term of challenge stressors and servant leadership, and that of hindrance stressors and servant leadership, were calculated, respectively. It is known from Model 4 in Table 3 that the interaction term of challenge stressors and servant leadership had a significant positive impact on harmonious passion (*B* = 0.16, *P* < 0.001), indicating that servant leadership significantly enhanced the positive relationship between challenge stressors and harmonious passion. Thus, Hypothesis 5 is supported.

**FIGURE 1:**
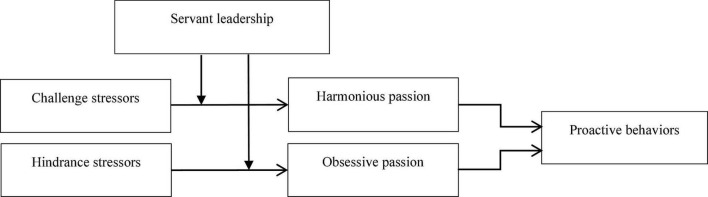
The overall theoretical model.

To explain the moderating effect more clearly, servant leadership was categorized into two groups representing high and low degrees, respectively (see Figure 1). When the degree of servant leadership was low, the impact of challenge stressors on harmonious passion was *B* = –0.13, *P* < 0.05. When the degree of service-oriented leadership was high, the impact of challenging stressors on harmonious passion was *B* = 0.24, *P* < 0.001.

**FIGURE 2:**
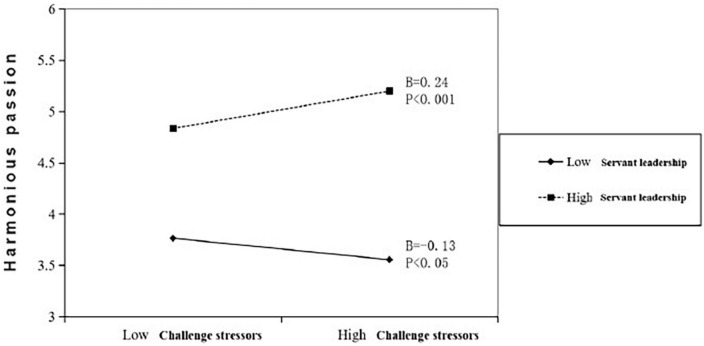
The moderating effect of servant leadership on the relationship between challenge stressors and harmonious passion.

As shown in Model 4 in Table 5, the interaction term of hindrance stressors and servant leadership had a significant positive impact on obsessive passion (*B* = 0.12, *P* < 0.01), indicating that servant leadership significantly enhanced the positive relationship between hindrance stressors and obsessive passion. Thus, Hypothesis 6 is not supported. This manuscript divides servant leadership into two groups with high and low degrees and presents Figure 2 to illustrate the moderating effect more clearly. As shown in Figure 2, when the degree of servant leadership was low, the impact of hindrance stressors on obsessive passion was *B* = 0.23, *P* < 0.05; when the degree of servant leadership was high, the impact of hindrance stressors on obsessive passion was *B* = 0.48, *P* < 0.001.

#### Test of the moderated mediating effect

To test whether the mediating effect of harmonious passion and obsessive passion was moderated by servant leadership, this study used Model 7 of the PROCESS plug-in of SPSS. The results were as follows.

When the degree of servant leadership was low, the mediating effect of harmonious passion was invalid [–0.02, 0.003]. When the degree of servant leadership was high, the mediating effect size was 0.02 and the confidence interval was [0.004, 0.028]. Also, the moderated mediating effect size was 0.01, and the confidence interval was [0.003, 0.018]. That is, the higher the degree of servant leadership, the stronger the mediating effect of harmonious passion was between challenge stressors and proactive behaviors. Hypothesis 7 is therefore supported.

When the degree of servant leadership was low, the mediating effect size of obsessive passion was –0.01 and the confidence interval was [–0.03, –0.001]. When the degree of servant leadership was high, the mediating effect size was -0.02 and the confidence interval was [—0.04, –0.004]. Also, the moderated mediating effect size was –0.01, and the confidence interval was [–0.01, 0.001]. Thus, Hypothesis 8 is not supported.

## Discussion

### Research findings

This study aims to further our understanding of the proactive behaviors and, specifically, to resolve previous inconsistencies in findings on the stressors–proactive behaviors relationship by distinguishing between challenge and hindrance stressors. Based on the research results and SDT, this study finds the following: (1) The challenge stressors and hindrance stressors of insurance salespeople had different effects on proactive behaviors. Namely, challenge stressors positively influenced proactive behaviors, while hindrance stressors negatively influenced proactive behaviors. (2) Harmonious passion and obsessive passion played mediating roles between challenge stressors and proactive behaviors, and between hindrance stressors and proactive behaviors. (3) Servant leadership positively moderated the relationship between challenge stressors and harmonious passion to a significant degree, and harmonious passion has been proven to have a significant positive correlation with proactive behaviors. Unexpectedly, servant leadership did not moderate the relationship between hindrance stressors and obsessive passion.

Considering the specific compensation structure in the insurance industry, prior researchers have paid considerable attention to improving performance-review criteria and creating incentives to spur employee proactivity (e.g., Wu, 2019; Lyons, 2020). However, our results show that whether salesperson view contextual factors as challenge and hindrance plays a significant role of their proactivity. The results warn of the risks that when salespeople appraise incentives as hindrance stressors, they may generate unmanageable feelings and obsessive passion, which can muzzle their proactive behaviors. In addition, since insurance sales position has high job requirements in nature (Grandey et al., 2013), which are typically deemed as work stressors, clearing the effect of stressors on proactive behaviors may go beyond the focus on criteria and incentives. This finding, which may go against practitioners’ intuitiveness and management mentality, provides a fresh perspective on the management of insurance salespeople.

### Theoretical contribution

First, this study fills important gaps in the literature as the first to simultaneously model the relationship between challenge and hindrance stressors and proactive behaviors through passion. Given the proved importance of examining stressors-related outcomes while controlling for both dimensions of stressors (Cavanaugh et al., 2000; Wallace et al., 2009), empirical research examining challenge and hindrance stressors with behavioral outcomes, however, is still quite sparse. This study, by distinguishing two categories of stressors based on CHM, provides integrated model of the impact of life insurance salespeople’s work stressors on proactive behaviors. Hence, our study avoids two main drawbacks of the previous studies: (1) the stressors were not classified, with one-sided emphasis on the negative or positive effect of stress; (2) although influences of different stressors were differentiated, the conclusions were directly reached without consideration of the internalization mechanism of external motivation before the stress caused employees to take a certain action.

Second, this manuscript offers an in-depth analysis of different passions inspired by different stressors. While researchers have called for a clearer articulation of the motivational mechanisms among the relationships between environments and proactive behaviors (e.g., George, 2007; Parker et al., 2010), previous studies have largely ignored the role of passion, which is portrayed as motivational constructs with superior quality (Liu et al., 2011). This study, by employing SDT, analyzes different work stressors in a more comprehensive way as related to the psychological processing behind passion emergence. By clarifying the mechanism of how insurance salespeople react to work stressors, this study introduces that passion can play a significant role in translating stressors into proactive behaviors, thereby enriching our understanding of proactive behaviors and SDT.

Third, this manuscript expands the boundary conditions under which work stressors affect the generation of work passion and examines the moderating role of servant leadership between work stressors and work passion. To address the call to explore more boundary conditions among the roles of environmental antecedents in proactivity studies (e.g., Bindl and Parker, 2011; Cai et al., 2019), we introduce servant leadership as a moderator, crystalizing how it influences employees’ passion emergence reacting to specific work stressors. This study expands our knowledge by affirming that servant leadership can amplify the positive effects of challenge stressors on harmonious passion and then contribute to employee proactive behaviors.

### Practical implications

The conclusions of this study have some specific implications for the daily operation of insurance companies:

First, insurance companies should train salespeople to view stressors as challenges far more often than hindrances. There is a wide range of stressors that may be deemed as hindrances, including low social status of the job, lack of customer resources, poor relationship between superiors and subordinates, suppression by team leaders, and unsupportive family members—all of which may impede employees’ proactive behaviors. Therefore, insurance companies should enhance the overall social image of the insurance industry through image promotion, etc., provide salespeople with customer resources through more channels, build a harmonious and fair working environment, formulate a basic law to limit the power of supervisors and provide channels for salespeople to complain and solve problems, and organize family gatherings regularly to help salespeople gain support from their families. In short, the hindrance stressors salespeople may encounter should be checked and eliminated one by one according to their order of importance.

Second, insurance companies should stimulate harmonious passion in salespeople and suppress obsessive passion. According to our findings, many behaviors of salespeople under the excessive pressure of performance appraisal, such as misleading sales, non-compliant rebates, and instigation of insurance cancelation, may improve performance in the short term but are harmful to the long-term development of insurance companies. Therefore, we suggest that insurance companies reduce the one-sided KPI assessment and instead organize positive competitions, build an honor recognition system for outstanding personnel, and convert more commissions into rewards and incentives, so as to stimulate challenge stressors among salespeople, encourage them to accept the challenges, and generate harmonious passion. In addition, we advocate publicity for outstanding employees as examples to inspire other salespeople and to further enhance their own harmonious passion and desire for success. Finally, it is necessary to establish a harmonious concept of success for salespeople and to create a harmonious and diligent working atmosphere in which everyone is willing to help each other. Companies must establish a proper evaluation system for identifying outstanding salespeople and objectively evaluate and analyze the reasons for employees’ success or failure.

Third, insurance companies should vigorously advocate servant leadership. The traditional top–down management philosophy in China has led to a lack of servant leadership and inhibited the proactive behaviors of salespeople. Team leaders lack service awareness and skills, which makes it difficult for salespeople to find solutions from the organization when they face difficulties in business. Working individually, they cannot use their personal strengths, and a cohesive sales team is not built.

Therefore, insurance companies should vigorously promote servant leadership and encourage leaders to do the following two things. Firstly, team leaders must establish a sense of service and apply service throughout the daily work of the entire team. According to SDT, they must ensure the necessary material and social-emotional conditions for the sales team, so as to create an agglomeration effect where salespeople do the sales work together. Secondly, while team leaders strengthen their own service capabilities (including the capability of coaching on products and sales skills), they should also strengthen communication and cooperation within the team and with other teams. When their own service level is not high enough, they should be able to find more internal and external service resources for the team. This ability of leveraging is a manifestation of servant leadership.

### Limitations and future directions of research

This study explores the influence of challenge and hindrance stressors experienced by life insurance salespeople on their proactive behaviors through harmonious and obsessive passions from the framework of SDT. In spite of the strengths and contributions mentioned above, several limitations should be considered.

First, this study may have the risk of common method bias because all variables were measured by self-reports (Spector, 2019). According to the CHM and SDT, what we explicitly focus on is perceived environmental factors (e.g., challenge or hindrance stressors, servant leadership) instead of objective factors, due to the acknowledged awareness that people’s behaviors are influenced by their subjective perception of a situation rather than the objective situation itself. Moreover, self-reported measurement is valid for measuring psychological needs and motivation (Paulhus and Vazire, 2007; Chan, 2009). However, future research could adopt other empirical methods or devices, such as scenarios with wearable devices, to enrich the data source and reduce this bias.

Second, contrary to our expectation, the interaction term of hindrance stressors and servant leadership had a significant positive impact on obsessive passion, and the moderated mediation effect of the whole model was not significant. Lazarus and Folkman (1984) claimed that the way individuals react to contextual factors (e.g., stressors) can vary widely according to individual characteristics that affect the pattern in which the individuals appraise those factors. Therefore, it is reasonable to speculate that when life insurance salespeople simultaneously treat stressors as hindrances and perceive servant leadership, some of them feel encouraged and comforted, leading to less obsessive passion, while many more may be overwhelmed by guilt, leading to obsessive passion. Future studies could analyze this relationship in greater detail by taking into account individual characteristics and environmental factors.

Finally, our data were collected from the life insurance industry in China. Given the no-basic-salary compensation structure, life insurance salespeople could be deemed as a good example for investigating the relationship between work stressors, passion, and proactive behaviors. However, it is not clear whether our results can be generalized to other industries, specially to those industries with relatively stable income. Moreover, the results may vary across countries. Individuals usually appraise work stressors in a consistent manner due to their social understanding of a specific factor or phenomenon (Lepine et al., 2005). Future research could replicate the hypotheses in other industries or for different cultures. Cross-cultural research in this domain may offer a more comprehensive understanding.

## Data availability statement

The raw data supporting the conclusions of this article will be made available by the authors, without undue reservation.

## Author contributions

AW designed the study, collected the data, and wrote the draft. LZ revised the theoretical model and manuscript, and was responsible for literature review and hypothesis. FS contributed to the method and data analysis parts, and also helped in improving the theoretical framework. All authors contributed to the article and approved the submitted version.
